# Hyperthyroidism from autoimmune thyroiditis in a man with type 1 diabetes mellitus: a case report

**DOI:** 10.1186/1752-1947-5-277

**Published:** 2011-07-03

**Authors:** John K Amory, Irl B Hirsch

**Affiliations:** 1 Division of General Internal Medicine, Department of Medicine, University of Washington School of Medicine, 1959 NE Pacific Street, Seattle, WA 98195, USA; 2 Division of Metabolism, Endocrinology and Nutrition, Department of Medicine, University of Washington School of Medicine, 1959 NE Pacific Street, Seattle, WA 98195, USA

## Abstract

**Introduction:**

The presentation, diagnosis, clinical course and treatment of a man with hyperthyroidism secondary to autoimmune thyroiditis in the setting of type 1 diabetes mellitus has not previously been described.

**Case presentation:**

A 32-year-old European-American man with an eight-year history of type 1 diabetes mellitus presented with an unintentional 22-pound weight loss but an otherwise normal physical examination. Laboratory studies revealed a suppressed thyroid-stimulating hormone concentration and an elevated thyroxine level, which are consistent with hyperthyroidism. His anti-thyroid peroxidase antibodies were positive, and his thyroid-stimulating immunoglobulin test was negative. Uptake of radioactive iodine by scanning was 0.5% at 24 hours. The patient was diagnosed with autoimmune thyroiditis. Six weeks following his initial presentation he became clinically and biochemically hypothyroid and was treated with thyroxine.

**Conclusion:**

This report demonstrates that autoimmune thyroiditis presenting as hyperthyroidism can occur in a man with type 1 diabetes mellitus. Autoimmune thyroiditis may be an isolated manifestation of autoimmunity or may be part of an autoimmune polyglandular syndrome. Among patients with type 1 diabetes mellitus who present with hyperthyroidism, Graves' disease and other forms of hyperthyroidism need to be excluded as autoimmune thyroiditis can progress quickly to hypothyroidism, requiring thyroid hormone replacement therapy.

## Introduction

One percent of adults with type 1 diabetes mellitus will develop hyperthyroidism, with Graves' disease, multi-nodular goiters and toxic adenomas being the most common causes [1]. Infrequently, hyperthyroidism in type 1 diabetes mellitus can be secondary to autoimmune thyroiditis. Autoimmune thyroiditis initially presents with signs and symptoms of hyperthyroidism and serological evidence of thyroid autoimmunity, but without evidence of the immune-mediated thyroid stimulation characteristic of Graves' disease. As autoimmune thyroiditis often irreversibly damages the thyroid gland, this condition frequently progresses to hypothyroidism, requiring thyroid hormone replacement therapy [2]. In this report, we document the initial presentation, diagnosis, clinical course and treatment of a man with hyperthyroidism secondary to autoimmune thyroiditis in the setting of type 1 diabetes mellitus. We were unable to identify a similar case report in the medical literature.

## Case presentation

Our patient was a 32-year-old Caucasian man with an eight-year history of type 1 diabetes mellitus. At the time of his diabetes diagnosis, he was noted to be positive for both elevated levels of antibodies to glutamic acid decarboxylase at 64 U/mL (normal, less than 1.45 U/mL) and anti-islet cell antibodies with a titer of 1:256 (normal, less than 1:4), but was otherwise healthy. His thyroid function was normal. He had no family history of diabetes or other autoimmune disease. Over the past eight years, his diabetes had been treated with insulin by using a MiniMed Paradigm 722 insulin pump (Medtronic Inc., Northridge, CA, USA) as well as with pramlintide 15 μg three times daily prior to meals. His average blood glucose level was 150 mg/dL, and his hemoglobin A1c (HbA1c) was 7.5%. He had no evidence of retinopathy, neuropathy or nephropathy.

He presented for medical care with a complaint of unintended weight loss of 22 pounds, from 203 pounds (body mass index (BMI) 26.1) to 181 pounds (BMI 23.2), over a period of three months. His weight loss was not associated with abdominal pain, diarrhea, steatorrhea or a change in diet or exercise. He denied palpitations, heat intolerance, neck soreness or other symptoms referable to his thyroid. His physical examination revealed a well-developed, well-nourished man in no apparent distress. He had a normal blood pressure level of 130/80 mmHg, and his pulse was within the normal range at 72 beats/minute. There was no lid lag or proptosis. His thyroid gland was not enlarged and was non-tender. There was no cervical lymphadenopathy or appendicular tremor. The remainder of his physical examination was within normal limits.

His laboratory assessment revealed normal blood counts, electrolytes and liver and kidney function. His serum thyroid-stimulating hormone (TSH) level was suppressed at 0.033 mIU/L (normal range, 0.4 to 5.0 mIU/L), and his free thyroxine level was elevated at 33 pmol/L (normal range, 7.7 to 15.4 pmol/L). An anti-thyroid peroxidase antibody was elevated at 13.2SD (normal, < 2.0SD), and the results of a thyroid-stimulating immunoglobulin (TSI) assay were undetectable. His serum thyroglobulin was normal, arguing against thyroid hormone self-administration (thyrotoxicosis factitia) [3]. A radioactive iodine update scan was performed to exclude the possibility of TSI-negative Graves' disease. The uptake of radioactive iodine at four hours was 1.3% (normal, 4% to 20%) and 0.5% at 24 hours (normal, 10% to 30%), consistent with thyroiditis. Notably, his HbA1c improved to 6.9% without any change in his insulin regimen.

**Figure 1:**
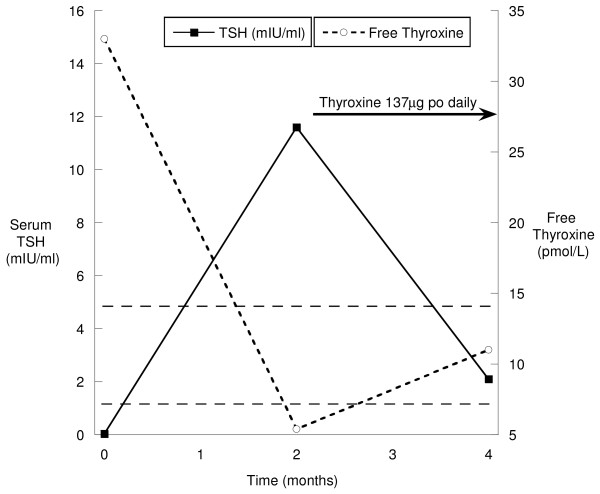
**Thyroid-stimulating hormone (TSH) and free thyroxine in a man with autoimmune thyroiditis**. Initially, our patient presented with hyperthyroidism (elevated thyroxine and suppressed TSH levels), which evolved to hypothyroidism (elevated TSH and low thyroxine levels) at month two. His thyroid indices normalized with thyroid hormone replacement therapy (indicated by arrow) four months after his initial presentation. The dotted lines represent the upper and lower limits of the normal ranges for TSH and free thyroxine.

The patient was diagnosed with autoimmune thyroiditis and instructed to watch carefully for signs or symptoms of hypothyroidism. Six weeks after his diagnosis he developed fatigue, cold intolerance and had regained all of the weight he had lost earlier. His TSH level was elevated to11 mIU/L, and his free thyroxine concentration was below the lower limit of the normal range at 5.4 pmol/L. Therefore, given the combination of his biochemistry and symptomatic hypothyroidism, the decision was made to initiate thyroid hormone replacement therapy at a dose of 1.5 μg/kg (137 μg daily for a 91 kg individual), which normalized his serum TSH and free thyroxine values and alleviated his symptoms of hypothyroidism (Figure 1). The patient has remained euthyroid on a stable dose of thyroxine for over 12 months without recurrence of his hyperthyroid symptoms. His glycemic control has returned to baseline values.

## Discussion

This case nicely illustrates the dynamic nature of thyroiditis with an initial hyperthyroid phase due to the release of pre-formed thyroid hormone from the injured thyroid gland followed by a hypothyroid phase due to glandular dysfunction. In addition to the usual symptoms of hyperthyroidism, our patient experienced a transient improvement in his glycemic control, as evidenced by a reduction in his HgA1C level early in his illness. In this patient, the diagnosis of autoimmune thyroiditis was made on the basis of the presence of anti-thyroid peroxidase antibodies and the absence of evidence of another etiology for hyperthyroidism, such as Graves' disease, multi-nodular goiter or toxic adenoma. As expected, six weeks after he presented and was diagnosed with hyperthyroidism, his serum thyroxine concentration fell below the normal range and he began to manifest symptoms of hypothyroidism, necessitating thyroid hormone replacement therapy.

The evaluation of a patient who presents with hyperthyroidism frequently includes a radioiodine uptake scan as well as testing for anti-thyroid antibodies, specifically thyroid-stimulating receptor antibodies and anti-thyroid peroxidase antibodies, to make the differential diagnosis between Graves' disease, multi-nodular goiter, toxic adenoma and thyroiditis. Distinguishing these conditions is desirable, as the treatment of these entities differs. Graves' disease, goiters and adenomas are treated with thionamide medications (such as methimazole or propylthiouracil), radioactive iodine or surgical resection. In contrast, thyroiditis can be managed prospectively, as many patients with this disease return to normal over time or develop hypothyroidism, as was the case in our patient. An alternative approach to treating a patient such as the one described in this report could have been the empirical use of a thionamide medication, without the use of scanning or testing for anti-thyroid antibodies, as therapy for a presumptive diagnosis of Graves' disease. This approach would save some money in terms of diagnostic testing but could lead to unnecessary medication-induced side effects or greater hypothyroidism six to eight weeks later at follow-up. To our knowledge, a formal risk-benefit analysis comparing these diagnostic approaches has not been published in the medical literature. With either approach, the correct diagnosis of thyroiditis in our patient would have been made when he re-presented at follow-up with symptoms of weight gain, hypothyroidism and an elevated TSH level. Therefore, the risk of initial misdiagnosis based on the conservative testing strategy is not great, unless the patient were to receive radioactive iodine or were to experience an unnecessary side effect of methimazole treatment.

Evidence of thyroid autoimmunity is common in individuals with type 1 diabetes mellitus, with up to 27% of young adults with type 1 diabetes mellitus having detectable titers of anti-thyroid peroxidase antibodies [4]. Many of these individuals eventually manifest clinical thyroid disease, which is almost always hypothyroidism. It is uncommon for these patients to present with symptomatic signs and symptoms of hyperthyroidism, which may be mild enough to be asymptomatic or could be missed by clinicians. Symptomatic thyroid disease is more common in women but can occasionally occur in men, as demonstrated by our present case. Furthermore, clinically evident thyroid disease is more common in children with antibodies to glutamic acid decarboxylase [5], suggesting a common immune pathophysiology between the development of type 1 diabetes mellitus and autoimmune thyroid disease. Indeed, some individuals may develop other manifestations of autoimmunity, such as adrenal dysfunction, celiac disease or pernicious anemia, due to an underlying autoimmune polyglandular syndrome [6]. To exclude the possibility of an autoimmune polygladular syndrome, our patient underwent testing for pernicious anemia and celiac disease, but no evidence of either condition was found. The most common form of autoimmune polyglandular syndrome is associated with specific haplotypes of class II human leukocyte antigen alleles, but this test is not commonly performed in a clinical setting. It is possible that the co-occurrence of autoimmune thyroiditis and type 1 diabetes mellitus in our patient was coincidental; however, the elevated risk of other types of autoimmune diabetes in individuals with type 1 diabetes mellitus suggests that a causal link between these conditions is more likely than not.

The effect of hyperthyroidism on diabetes is complex [7]. Hyperthyroidism stimulates increased metabolism in many tissues, leading to an increased demand for glucose [8]. This in turn triggers stimulation of gluconeogenesis and lipolysis, leading to weight loss. Overall, the increased uptake of glucose from the circulation, coupled with reductions in weight, can result in improvements in markers of glycemic control such as those we observed in our patient. Conversely, hypothyroidism appears to increase insulin resistance independently of weight gain [9,10] and has been associated with an increased risk of symptomatic hypoglycemia [11]. Protracted hypoglycemia can lead to increases in weight, which exacerbate insulin resistance and worsen glycemic control.

## Conclusion

In conclusion, in this case report, we have described the presentation, diagnosis, clinical course and treatment of a man with hyperthyroidism secondary to autoimmune thyroiditis in the setting of type 1 diabetes mellitus. Clinicians caring for patients with diabetes should be aware of hyperthyroidism secondary to autoimmune thyroiditis so that they can distinguish it from other, more common forms of hyperthyroidism, such as Graves' disease, toxic adenomas or multi-nodular goiters. In a patient with type 1 diabetes mellitus such as ours, the most common etiology is Graves' disease. Many clinicians would not have considered obtaining such a comprehensive work-up, including a radioactive iodine uptake, in this setting. However, a thorough evaluation was essential in our patient, as the diagnosis of thyroiditis prevented unnecessary treatment with medications, radioactive iodine or surgery. Indeed, the treatment strategy was completely altered when it was determined that the etiology of our patient's hyperthyroidism was autoimmune thyroiditis, and there was no indication for the use of anti-thyroid medications.

### Patient's perspective

"My initial symptoms were weight loss, highly increased energy level, and lack of sleep (also a lack of feeling the need for sleep). Anecdotally, during this time (roughly one month leading up to diagnosis) I worked my normal nine to 12 hour days and then spent an additional four to eight hours in the evenings remodeling my kitchen. I did this without undue feelings of exhaustion. When the hyperthyroidism finally slowed down, it was remarkable how quickly I transitioned to hypothyroidism. As I remember it, in the course of a week or two I went from having a healthy, controlled energy level to sleeping 10 hours, waking up still exhausted, and exhibiting symptoms of mild depression. The change in symptoms was both stark and rapid. As the hypothyroidism presented, my doctors were able to quickly get me to a balanced dose of thyroxine, and my energy levels have been in a good place for the last year."

### Consent

Written informed consent was obtained from the patient for publication of this case report and any accompanying images. A copy of the written consent is available for review by the Editor-in-Chief of this journal.

## Competing interests

The authors declare that they have no competing interests.

## Authors' contributions

JKA and IBH both cared for the patient as primary care provider and endocrinologist, respectively. Both authors contributed to the drafting of the article, and both approved the final manuscript.
